# An emotion regulation role of ventromedial prefrontal cortex in moral judgment

**DOI:** 10.3389/fnhum.2014.00873

**Published:** 2014-10-28

**Authors:** Chuanpeng Hu, Xiaoming Jiang

**Affiliations:** ^1^Department of Psychology, School of Social Science, Tsinghua UniversityBeijing, China; ^2^School of Communication Sciences and Disorders, Faculty of Medicine, McGill UniversityMontréal, QC, Canada

**Keywords:** vmPFC, moral judgment, fMRI, lesion study, emotion regulation

Moral dilemma is an effective approach to the investigation of neural mechanisms underlying moral judgment. A typical dilemma (e.g., the “footbridge dilemma”) describes a hypothetical situation in which a protagonist is faced with two exclusive options: killing one person to save more lives (a “utilitarian” judgment to maximize the utility), or doing nothing and watching those people die (a deontological judgment adhering strictly to the ethic rule of “do not kill”). Neuroimaging (Greene et al., 2001, 2004) and lesion studies (e.g., Koenigs et al., 2007) have identified the ventromedial prefrontal cortex (vmPFC) as a critical brain structure in resolving the moral dilemma. However, the precise function of vmPFC is still underspecified (Greene, 2007; Moll and De Oliveira-Souza, 2007).

Recently, Shenhav and Greene (2014) proposed an integrative judgment theory of vmPFC. To dissociate the mechanisms of different mental processes in moral judgment, they asked the participants to make one of the three types of judgment when facing a moral dilemma: an emotional assessment (EA), in which the aversive-ness of both options were assessed, or a utilitarian assessment (UA), in which the utility value of both options were assessed, or an integrative moral judgment (IMJ), in which the overall acceptability of each option was judged. The vmPFC was more activated in the IMJ condition than in the other two conditions. Also, in contrast to the activation of amygdala, which demonstrated a negative correlation with the frequency of utilitarian judgment in IMJ, the vmPFC activation was positively correlated with that frequency. These results led Shenhav and Greene to conclude that the role of vmPFC in moral judgment is “to integrate disparate value signals into a more abstract, summary value representation.”

Still, the integrative judgment theory cannot easily accommodate the findings in vmPFC-damaged patients: If vmPFC only serves to integrate the emotional signal from amygdala and other regions, then damage to the vmPFC would allow the aversive emotion to affect the moral judgment directly, leading to more deontological choices. This prediction contradicts previous findings that vmPFC damaged patients preferred the utilitarian option (Koenigs et al., 2007) and did not generate emotional responses when endorsing a utilitarian judgment (Moretto et al., 2010).

This discrepancy could be resolved from an emotion regulation perspective of vmPFC, which proposes that vmPFC recursively appraises (or reappraises) the affective meaning of moral events in making moral judgment. The vmPFC generates an affective meaning to a utilitarian option and re-codes the meaning in accordance with the current goal/context (Roy et al., 2012). The emotion regulation view agrees with the integrative judgment theory (Shenhav and Greene, 2014) that vmPFC receives the emotional signal from amygdala and other brain regions when making moral judgment. Moreover, this view extends integrative judgment theory in two critical ways.

Firstly, the emotion regulation view proposes that vmPFC plays a critical role in generating social emotion to an option according to the general moral principle; while the integrative judgment theory does not predict an affective meaning generation process or how vmPFC is involved in such a process. Consistent with this prediction, an fMRI study demonstrated that vmPFC was only responsive to moral stimuli but not to non-moral emotional stimuli (Moll et al., 2002). In moral judgment, Shenhav and Greene (2014) found the most enhanced connectivity between vmPFC and amygdala under the EA condition.

According to the emotion regulation view, when facing a moral dilemma, the protagonist does not have a Pavlovian emotional response to the utilitarian option, instead, the vmPFC appraises this option against the general moral principle (e.g., “do not kill innocent lives”) and interacts with amygdala to generate an aversive emotion to this option. Mal-function of vmPFC causes failure to generate appropriate moral emotion, which explains why these patients were affectively blunted (Anderson et al., 2006) and showed fewer emotional responses on facing moral dilemma (Moretto et al., 2010). As a result, the utility-calculation process takes charge, leading to more utilitarian decisions (Koenigs et al., 2007).

Secondly, the emotion regulation view hypothesizes that vmPFC re-appraises the initial affective meaning of the utilitarian option against the current situation/goal (e.g., requiring all things to be considered), thus altering the strength/valence of the emotion; while the integrative judgment theory does not specify how emotional meaning is recomputed after the integration. In moral dilemmas, the re-appraisal of the utilitarian option, which is subserved by vmPFC, weakens the negative emotion toward this option and leads to a more positive attitude toward a utilitarian response. Indeed, a positive correlation between vmPFC activation and the frequency of utilitarian choice was shown in the IMJ condition (Shenhav and Greene, 2014), as well as a negative correlation between the utilitarian response and negative emotion toward that response (Moretto et al., 2010).

The emotion regulation view also explains why vmPFC-damaged patients reject more unfair offers in the ultimatum game. Under this view, the unfair offers elicit a negative emotion to both healthy controls and vmPFC-damaged patients. The vmPFC functions to re-appraise the unfair offers in light of the potential gain, re-coding the negative emotion to a more positive direction and consequently increasing acceptance of unfair offers; while the damage of vmPFC causes failure to re-appraise the unfair offers and results in more rejection of unfair offers (Koenigs and Tranel, 2007). The emotion regulation view can apply to the real-world dilemma when the moral (social) standard has to be violated because of a positive consequence of an action (e.g., the son steals money for a sick parent). The reappraisal of negative affective meaning of moral violation may vary according to how positively the consequence of that action is evaluated, leading to different judgments.

## Conflict of interest statement

The authors declare that the research was conducted in the absence of any commercial or financial relationships that could be construed as a potential conflict of interest.
